# Psychological distress among Brazilian workers during the initial stage of the COVID-19 pandemic: a descriptive study

**DOI:** 10.3389/fpubh.2024.1283310

**Published:** 2024-02-19

**Authors:** Melissa Spröesser Alonso, Maria Cristina Pereira Lima, Adriano Dias, Juan Carlos Camacho-Vega, Juan Jesus García-Iglesias, Carlos Ruiz-Frutos, João Marcos Bernardes, Juan Gómez-Salgado

**Affiliations:** ^1^Public/Collective Health Graduate Program, Botucatu Medical School, São Paulo State University (UNESP), Botucatu, Brazil; ^2^Department of Architectural Construction II, Higher Technical School of Building Engineering, University of Seville, Seville, Spain; ^3^Faculty of Labour Sciences, Department of Sociology, Social Work, and Public Health, University of Huelva, Huelva, Spain; ^4^Safety and Health Postgraduate Programme, Universidad Espíritu Santo, Guayaquil, Ecuador

**Keywords:** COVID-19, psychological distress, sense of coherence, work engagement, workers, epidemiology

## Abstract

**Background:**

COVID-19 pandemic imposed drastic and abrupt changes to working environment and organization and that might have caused additional negative effects on mental health. Thus, this study aimed to quantify and assess the severity of psychological distress experienced by Brazilian essential and nonessential workers during the first months of the COVID-19 pandemic.

**Methods:**

This descriptive study included 2,903 participants who answered an online questionnaire between April and May 2020. The research questionnaire was translated and culturally adapted to the Brazilian population from a questionnaire developed and validated for the Spanish population. Variables were analyzed using simple and cumulative percentage distributions and measures of central tendency and dispersion. The Wilson score interval was used to calculate confidence interval (CI) for the main outcome, psychological distress.

**Results:**

It was observed a high prevalence (72.6%) of psychological distress among the study’s participants. They also presented a median risk perception score of 60 (out of a maximum of 90), and their greatest concern was transmitting the virus to family members, close contacts or patients. Furthermore, it was found a lower sense of coherence and work engagement among the participants than those observed in previous studies conducted in other countries.

**Conclusion:**

Almost three quarters of the study’s participants were classified as presenting psychological distress. Thus, it is imperative to provide mental health remotely delivered interventions to workers during public health events that require prolonged social distancing measures.

## 1 Introduction

A pandemic event is an extraordinary phenomenon that has major implications in many life domains, such as physical and psychological well-being, working experience, social life, economic status, leisure activities, and others. The coronavirus disease 2019 (COVID-19) pandemic - which evolved from a cluster of a novel viral pneumonia in Wuhan, China, reported on the last month of 2019, to a full pandemic scenario on 11 March 2020 (1) - was not different, since it caused significant health, social, educational, working and economic burden all around the world (2–8). In Brazil, specifically, the COVID-19 pandemic precipitated a multifaceted crisis, encompassing hundreds of thousands of deaths, reduced economic activity, decreased exports, an important surge in unemployment, increased precarious working conditions, a strong contraction of the National Gross Domestic Product, food-insecurity intensification, an increase in domestic violence and femicides, and political disputes, all of which led to an exacerbation of previous social disparities and inequalities (9). Together, this atypical situation, its related challenges and negative consequences have culminated in loneliness, insomnia, fear, grief (at various levels), anxiety and depression (10–12), jeopardizing mental and physical health all around the globe.

In Brazil, the first confirmed COVID-19 case was reported on 25 February 2020, in the city of São Paulo (13). The public universal health system (known as Sistema Unico de Saúde) already had shown the capacity to deal successfully with epidemics in the recent past (Influenza in 2009 and Zika in 2015, for example) (14). However, during the initial stage of the pandemic in Brazil, testing rates were extremely low, contact tracing was practically non-existent, epidemiological data was unspecific and not transparent (15–20). Consequently, between the first reported case and the end of May, 2020, Brazil already recorded the third-highest number of confirmed COVID-19 infections globally (21), with 498,440 cases and 28,834 deaths (22). And, a year after the first COVID-19 case was diagnosed, Brazil became the global epicenter for COVID-19 (15–17).

Several studies have highlighted the significance of examining the impact of the COVID-19 pandemic on mental health within specific socio-cultural contexts. For instance, a study by Goularte et al. emphasized the need for region-specific analyses to understand the diverse experiences of individuals during the pandemic (23). Brazil, with its unique socio-economic and cultural characteristics, presents a compelling case for such focused research. Our decision to concentrate on Brazil is aligned with the recommendations of literature who emphasized the importance of considering country-specific factors, such as variations in healthcare systems, economic conditions, and cultural factors when investigating the mental health consequences of the pandemic (24, 25).

Not surprisingly, more than 60 and 55% of Brazilians had low or no confidence in the government (26, 27) and considered the country’s pandemic response to be inadequate and inefficient (27), respectively. This is concerning, since the perceived efficiency and trustworthiness of a government can influence its citizens’ well-being (28). Moreover, considering that trust in governmental institutions and the perception of an adequate governmental response is a determinant of mental health during public health emergencies (29–33), this situation becomes even more severe.

In addition to the issues discussed above, the COVID-19 pandemic also imposed drastic and abrupt changes to working environment and organization which might cause additional negative effects on mental health. Workers that were doing their work from home experienced reduced social interactions, decreased overall physical activity, inadequate workstations, inappropriate distractions and/or interruptions, blurred work-life boundaries, extended working hours and higher workload (34). While those workers whose work could not be done from home were subjected to an increased likelihood of infection, constant vigilance and the adoption of new demanding hygiene measures to avoid SARS-CoV-2 exposure and the fear of being infected and transmitting the infection to family members (35). Therefore, the variables studied were the usual sociodemographic ones such as sex, age, marital status, highest education level completed, Brazilian region of residence, number of children and health status among others. Some more specific ones that could affect stress levels were included, such as residence type, pet ownership, living with someone who has disability, etc. (36). In addition, other occupational variables such as occupational group, employment relationship, work arrangement, employer provided all materials and means necessary to work efficiently and to work safely, experienced more conflicts at work, experienced an increase in the workload, experienced more work stress, and current work satisfaction, all of them can cause a disturbance at work level that can influence stress levels (37, 38). And finally, as (mis)information and the level of knowledge about COVID-19, both too much and too little, can increase or decrease stress levels, variables in this regard are included such as: information sources, number of information sources used, clarity and accuracy of employer information regarding COVID-19, hours per day exposed to COVID-19 information, fact-checking, self-perceived COVID-19 transmission knowledge, self-perceived COVID-19 preventive measures knowledge, self-perceived COVID-19 symptoms knowledge, self-perceived COVID-19 prognosis knowledge; and self-perceived COVID-19 treatment knowledge (39–41).

Amidst these challenges, fostering a strong sense of coherence and promoting work engagement may be one path to enhance the overall capacity of workers to cope with the challenges imposed by a pandemic crisis, and in doing so protect their mental health. Sense of coherence is a construct that expresses a person’s ability to evaluate and understand a negative situation, to cope with it making use of adequate and available resources, and to perceive the situation as being worth of investing energy to overcome it rather than a burden that should be avoided (33, 42). While work engagement - characterized by vigor, dedication, and absorption in one’s work - can be considered a valuable resource for workers to cope with unstable scenarios generated by demanding and adverse circumstances (43). Likewise, it is known from previous studies that sense of coherence and work engagement are key influencing factors for workers (22) and that lower sense of coherence level may be a protective factor in later stages of the pandemic (44). Work engagement and sense of coherence positively correlated with each other and both negatively with psychological distress. So, workers, though experiencing psychological distress, perceive their work positively and satisfactorily despite the severity of the situation and the harsh working conditions (45).

Even though many studies about the mental health consequences of the pandemic have already been published, particularly among frontline healthcare workers, the complexity of the Brazilian pandemic context deserves investigation, as do those workers that were not in the frontline but also were exposed to many factors that affect mental health. However, there are no specific studies on the working population in Brazil, and it represents the 62.6% of total population (46). In addition to the total population it represents, the lack of studies on workers in any field, changes in their working conditions and the incidence of the disease in early stages, among other reasons, this study allows us to offer a context that has not been studied previously. Therefore, this study aimed to assess the prevalence of psychological distress experienced by Brazilian workers (essential and nonessential) during the early months of the COVID-19 pandemic, while also describing other participant’s characteristics and important aspects of their pandemic experience that may have had an impact on their psychological well-being, such as the sense of coherence and work engagement.

## 2 Methods

This descriptive study is part of a larger international project, coordinated from Spain, and carried out in 16 countries from Latin America, Europe, Africa, and Asia. In Brazil the research was authorized by the Brazilian National Research Ethics Committee (CAAE 30437120.4.0000.5411, 04/23/2020).

The period of data collection in Brazil was from April 23 to May 30, 2020. Given the crucial need for social distancing to control the COVID-19 pandemic, mobility difficulties due to lockdowns and other distancing measures, and to protect the research team, participants were recruited through invitations sent by email and advertisements in the press and social networking sites (WhatsApp, Facebook, Instagram, Twitter, and LinkedIn). Participation in the study was voluntary, there was no incentive or remuneration, and each participant signed a virtual informed consent before answering the research questionnaire. Using a snowball sampling with multiple entry points, respondents were asked to invite other potential participants to take part in the research after they completed the survey. Snowball sampling techniques are frequently employed in cases where the study population is unknown, presenting challenges in the selection of participants who satisfy the specified eligibility criteria (47). Thus, since there is not a national registry of all Brazilian workers, due to the high percentage of informal workers (in 2019 41.6% of Brazilian workers were informal), snowball sampling was deemed a suitable method for the recruitment of study participants. It is also worth noting that, even though healthcare workers are not considered a “hard-to-reach” population, the pandemic introduced challenges such as healthcare workers stigmatization, frequent work site changes, as well as sick leaves due to suspected COVID-19 infection, making it difficult to identify and access these workers. Recognizing snowball sampling’s ability to facilitate access and encourage participation, the research team chose this method for efficient identification and recruitment of participants working on the pandemic frontline. In addition, the total population size in Brazil is 203,080,756, according to the Brazilian Statistical Institute of Geography and Statistics (46), of which 6.2% are formally employed Taking as a reference the total sample size of formally employed people, which amounts to 12,591,006, the sample size required is 239 subjects, with a confidence level of 95%, a proportion of 5%, and a 15% sample failure rate expected.

The research questionnaire, Emotional Impact Questionnaire COVID-19 Brazil (EIQ-BR), was translated and culturally adapted to the Brazilian population from a questionnaire developed and validated for the Spanish population. Detailed information on the Spanish questionnaire development and validation is described elsewhere (47). EIQ-BR translation procedure followed Beaton’s recommendations to translate and cross-culturally adapt questionnaires (48). Thus, EIQ’s Spanish version was initially translated to Brazilian Portuguese by two translators, after that a synthesis of both translations was conducted, this version was then translated back to Spanish. Since the back translated questionnaire and the original version agreed, the questionnaire was examined by 10 academic experts for face and content validity, item relevance and comprehensibility. In response to the judges’ feedback, changes were made accordingly, and the questionnaire’s comprehensibility was tested in a pilot survey on a sample size of 20 Brazilian workers.

EIQ-BR was made available online at (https://cutt.ly/IMPACT_COVID-19_BRASIL) and open to anyone interested in responding. Thus, inclusion criteria for this study - residing in Brazil during the pandemic, to be working at the time of enrolment and being 18 years of age or older - were applied after questionnaire completion and resulted in a total of 2,903 participants.

To enhance survey completion rates a progress bar was incorporated into the questionnaire to display participants’ progression throughout the survey and the total number of questions was limited to less than 200 (49). Hence, the questionnaire comprised 147 questions divided into 11 sections: sociodemographic characteristics, occupational profile, health-related characteristics, COVID-19 knowledge, COVID-19 contact history, COVID-19 perceived symptoms, COVID-19 risk perception, preventive measures, sense of coherence, work engagement and psychological distress.

In this study, the following sociodemographic variables were selected for analyses: sex (male and female); age (complete years); marital status (single, married or living with a partner, separated/divorced and widowed); highest education level completed (high school, bachelor, specialization, master’s degree and PhD); Brazilian region of residence (North, Northeast, Midwest, Southeast and South); residence type (apartment with balcony, apartment without balcony, house with backyard, house without backyard and other); children; pet ownership; living with someone who has physical disability; living with someone who has intellectual disability; and living with someone who has visual or auditive or multiple disabilities (the last five questions had “yes or no” answers).

The occupational variables were major occupational group (white-, blue-, pink-collar and others - white-collar included scientists, artists, executive workers, administrative workers, managerial workers, and technicians; blue-collar included farmers, foresters, fishermen, workers in production of industrial goods and services, repair workers and maintenance workers; pink-collar included service sector workers; and others included military personnel, police officers, firefighters and other occupations not included in the Brazilian Occupation Classification Index); healthcare professional (yes and no); employment relationship (self-employed, civil servant and private sector employee); work arrangement (part-time at home, part-time not at home, full-time at home, full time not at home and mixed); employer provided all materials and means necessary to work efficiently; employer provided all materials and means necessary to work safely (the last two questions had 1 through 10 scale answers, where 1 means “disagree completely” and 10 means “agree completely”); experienced more conflicts at work; experienced an increase in the workload; experienced more work stress (the last three questions had 1 through 10 scale answers, where 1 means “definitely not” and 10 means “definitely yes”); and current work satisfaction (1 through 10 scale, where 1 means “completely dissatisfied” and 10 means “completely satisfied”).

The health-related variables were self-perceived health status during the last 14 days (very good, good, fair, poor, and very poor); self-identifying as having a disability; self-identifying as having a chronic disease; self-reporting medication use; self-reported health care utilization in the past 14 days; and self-reported hospitalization history in the last 14 days (the last five questions had “yes or no” answers).

Regarding COVID-19 knowledge, the following variables were analyzed: information sources (official sources, television, radio, newspapers, social media, friends and family, others - official platforms include websites of official institutions or scientific societies; others includes Google and/or other search engines, scientific articles and other sources of information) number of information sources used (one, two, three, four, five, six and seven); clarity and accuracy of employer information regarding COVID-19 (1 through 10 scale, where 1 means “completely dissatisfied” and 10 means “completely satisfied”); hours per day exposed to COVID-19 information (up to 1 h, >1 up to 4 h, >4 up to 8 h and > 8 h); fact-checking (yes and no); self-perceived COVID-19 transmission knowledge; self-perceived COVID-19 preventive measures knowledge; self-perceived COVID-19 symptoms knowledge; self-perceived COVID-19 prognosis knowledge; and self-perceived COVID-19 treatment knowledge (the last five questions had 1 through 10 scale answers, where 1 means “insufficient” and 10 means “sufficient”).

EIQ-BR also presented five questions about basic COVID-19 knowledge (incubation period, symptoms, need for isolation after a positive test, form of transmission, and period of transmission), each of these questions had a possible answer of “yes,” “no,” or “I do not know.”

For its part, COVID-19 contact history variables were: living with a family member that has been infected (yes, no and have not had an infected family member); any co-worker was infected; close contact (more than 15 min or less than two meters away) with confirmed infected person; casual contact with confirmed infected person; any type of contact with people or materials suspected of being infected (the last four questions had “yes, no and do not know” as possible answers); and tested for COVID-19 (yes and no).

In addiction EIQ-BR collected data on perceived COVID-19 symptoms (cough, shortness of breath, fever, sore throat, rhinitis, chills, headache, myalgia, dizziness, and diarrhea) over the last 14 days. Therefore, two variables were analyzed: presented at least a symptom in the last 14 days (yes and no); and number of symptoms presented in the last 14 days (none, one, between two and four, between five and seven and between eight and ten).

Likewise, EIQ-BR had nine questions about COVID-19 risk perception. Each of these questions had a scale from 1 to 10 (where 1 means “not worried at all” and 10 means “very worried”) as an answer. Therefore, a risk perception score (discrete variable with a minimum of 9 and a maximum value of 90) was created by summing the score for each question. Other variables regarding risk perception were: self-perception of work as a risk for COVID-19 infection; belief that had contact with clients/patients that were a risk factor for COVID-19 transmission (this variable was analyzed only within the subset of participants who were not working remotely at the time of the survey); acceptance of COVID-19 infection as an occupational hazard (the last three questions had 1 through 10 scale answers, where 1 means “definitely not” and 10 means “definitely yes”); belief of avoidance from friends and/or relatives due to working in a high infection risk environment (this variable was analyzed only within the subset of participants who were not working remotely at the time of the survey); and belief that may have contracted COVID-19 (the last two questions had “yes, no and do not know” as possible answers).

Furthermore EIQ-BR gathered data about preventive measures. Questions with five answer choices, categorized from never to always, were used to identify the frequency of the following preventive behaviors: using the elbow to cover the mouth while sneezing or coughing; avoiding sharing eating utensils during meals; washing hands with soap and water; washing hands with hydroalcoholic solution; washing hands immediately after touching the nose, sneezing or coughing; washing hands after touching potentially contaminated objects; wearing face mask regardless of symptoms presence; keeping a distance of at least a meter and a half from others. The answers to these questions were converted to a numerical scale from 1 (never) to 5 (always) and a preventive behaviors scale was created by summing and then dividing by 8, the scores on the eight questions. Other variables regarding preventive measures adoption were also assessed: home confinement (fully, partially, and not confined); social distancing from friends and/or relatives due to perceived higher-risk work environment (yes and no - this variable was analyzed only within the subset of participants who were not working remotely at the time of the survey); and preventive measures effectiveness perception (1 through 10 scale, where 1 means “not effective at all not” and 10 means “very effective”).

To evaluate participant’s sense of coherence EIQ-BR made use of the Brazilian Portuguese version of the 13-item Sense of Coherence Scale (SOC-13) (50). The SOC-13 is a self-administered scale, which consists of 13 items, scored on a seven-point frequency Likert scale, and divided into three domains (comprehensibility, manageability, and meaningfulness) (51). The total scale score was obtained by the sum of the 13 item scores; and ranged from 13 to 91 points. The higher the score, the stronger the sense of coherence. Cronbach’s alpha as a measure of the internal consistency for the SOC-13 Brazilian Portuguese version entire scale was 0.81 (50).

Regarding work engagement, EIQ-BR made use of the Brazilian Portuguese version of the 9-items Utrecht Work Engagement Scale (UWES-9) (52). The UWES-9 is a self-administered scale, comprised of 9 items, scored on a seven-point frequency Likert scale, and divided into three domains (vigor, dedication, and absorption). Cronbach’s alpha as a measure of the internal consistency for the UWES-9 Brazilian Portuguese version entire scale was 0.94 (52). UWES-9 total score was obtained by the 9 item scores mean value and ranged from 0 to 6 points. The higher the score, the stronger the work engagement.

Finally, to evaluate psychological distress the EIQ-BR made use of the Brazilian Portuguese version of the 12-items General Health Questionnaire (GHQ-12) (53). The GHQ-12 is a self-administered screening instrument that evaluates psychological well-being and detects non-psychotic psychiatric disorders (54). Each item has four options, options 1 and 2 are worth zero points while options 3 or 4 are worth 1 point. For this study, a cut-off point of 3 was established, considering the presence of psychological distress in subjects with scores greater than or equal to 3. Cronbach’s alpha as a measure of the internal consistency for the GHQ-12 Brazilian Portuguese version entire scale was 0.88 (53).

The above variables were analyzed using simple and cumulative percentage distributions and measures of central tendency and dispersion (for discrete and continuous variables, respectively). The Wilson score interval was used to calculate confidence interval (CI) for the main outcome, psychological distress. Data analysis was conducted using the IBM Corp. Released 2019 SPSS Statistics for Windows (Version 26.0, IBM Corp., Armonk, NY). While missing data were estimated by chained equations multiple imputation using the “mice” function (package “mice,” R 4.1.1) adopting the predictive mean matching method (55, 56). The following variables had missing values (numbers in parentheses are the number of participants for whom data was missing): age (1); living with someone who has physical disability (311); living with someone who has intellectual disability (327); living with someone who has visual or auditive or multiple disabilities (260); COVID-19 incubation period knowledge (166); COVID-19 symptoms knowledge (166); COVID-19 need for isolation after positive test knowledge (166); COVID-19 form of transmission knowledge (166); COVID-19 transmission period knowledge (166); belief that had contact with clients/patients that were a risk factor for transmission (346); belief of avoidance from friends and/or relatives due to working in a high infection risk environment (240); and social distancing from friends and/or relatives due to perceived higher-risk work environment (240).

## 3 Results

Among the 2,903 participants, 73.0% were women and 34.1% reported having a chronic disease. The majority had more than high school education (90.0%) and were white-collar workers (75.7%). Almost half were civil servants (47.2%) and were working full-time at home (41.3%). Tables 1–3 present sociodemographic characteristics, occupational profile, and health-related information of the study participants.

**Table 1:** Participant’s sociodemographic characteristics (*n* = 2,903).

Variables	Percentage or median (IQR)
Sex	
Male	27.0%
Female	73.0%
Age	38.0 (18.0)
Marital status	
Single	35.2%
Married or living with a partner	54.5%
Separated/Divorced	9.4%
Widowed	0.9%
Children	
Yes	46.2%
No	53.8%
Pet ownership	
Yes	60.9%
No	39.1%
Highest education level completed	
High school	10.0%
Bachelor	26.5%
Specialization	28.0%
Master	19.2%
PhD	16.3%
Brazilian region of residence	
North	1.0%
Northeast	5.0%
Midwest	8.0%
Southeast	74.3%
South	11.7%
Residence type	
Apartment with balcony	21.4%
Apartment without balcony	16.7%
House with backyard	50.8%
House without backyard	7.2%
Other	3.9%
Living with someone who has physical disability	
Yes	3.0%
No	97.0%
Living with someone who has intellectual disability	
Yes	3.5%
No	96.5%
Living with someone who has visual or auditive or multiple disabilities	
Yes	12.1%
No	87.9%

**Table 2:** Participant’s occupational characteristics (*n* = 2,903).

Variables	Percentage or median (IQR)
Major occupational group	
White-collar	75.7%
Blue-collar	2.9%
Pink-collar	3.8%
Others	17.6%
Healthcare professional	
Yes	39.6%
No	60.4%
Employment relationship	
Self-employed	21.4%
Civil servant	47.2%
Private sector employee	31.4%
Work arrangement	
Part-time at home	16.4%
Part-time not at home	13.2%
Full-time at home	41.3%
Full time not at home	22.5%
Mixed	6.5%
Employer provided all materials and means necessary to work efficiently	8.0 (5.0)
Employer provided all materials and means necessary to work safely	9.0 (4.0)
Experienced more conflicts at work	3.0 (6.0)
Experienced an increase in the workload	7.0 (8.0)
Experienced more work stress	8.0 (6.0)
Current work satisfaction	6.0 (4.0)

**Table 3:** Participant’s health-related characteristics (*n* = 2,903).

Variables	Percentage or median (IQR)
Self-identifying as having a disability	
Yes	4.3%
No	95.7%
Self-identifying as having a chronic disease	
Yes	34.1%
No	65.9%
Self-reporting medication use	
Yes	44.9%
No	55.1%
Self-perceived health status during the last 14 days	
Very good	32.8%
Good	48.4%
Fair	16.0%
Poor	2.4%
Very poor	0.4%
Self-reported health care utilization in the past 14 days	
Yes	7.5%
No	92.5%
Self-reported hospitalization history in the last 14 days	
Yes	0.7%
No	99.3%

Regarding COVID-19 knowledge, 72.1% of the participants answered correctly all five questions. More than half of the participants were exposed to COVID-19 information for up to 4 h (70.6%) and, even more impressive, 94.0% declared engaging in fact-checking behavior. In relation to COVID-19 symptoms and contact history, 47.8% reported presenting between two and four COVID-19 symptoms during the previous 14 days and, even though, 7.4% reported that a family member had been infected only 4.6% had been tested for COVID-19. Transmitting the virus to family members, close contacts or patients was the participants’ greatest concern; it is also interesting to note that the participants perceived the preventive measures as being very effective. Tables 4–6 present participants’ COVID-19 knowledge, contact history, symptoms, risk perception and adhesion to preventive measures.

**Table 4:** Participants’ COVID-19 knowledge (*n* = 2,903).

Variables	Percentage or median (IQR)
Information sources	
Official sources	65.1%
Television	67.5%
Radio	20.1%
Newspapers	62.0%
Social media	80.8%
Friends and family	42.4%
Others	50.8%
Number of information sources used	
One	8.7%
Two	13.8%
Three	18.7%
Four	21.2%
Five	18.9%
Six	12.6%
Seven	6.1%
Clarity and accuracy of employer information regarding COVID-19	8.0 (4.0)
Hours per day exposed to COVID-19 information	
Up to 1 h	22.6%
>1 up to 4 h	48.0%
>4 up to 8 h	17.2%
>8 h	12.2%
Fact-checking	
Yes	94.0%
No	6.0%
Self-perceived COVID-19 transmission knowledge	9.0 (3.0)
Self-perceived COVID-19 preventive measures knowledge	9.0 (3.0)
Self-perceived COVID-19 symptoms knowledge	8.0 (3.0)
Self-perceived COVID-19 prognosis knowledge	7.0 (3.0)
Self-perceived COVID-19 treatment knowledge	6.0 (4.0)
COVID-19 incubation period basic knowledge question	
Correct answers	91.0%
Incorrect answers	9.0%
COVID-19 symptoms basic knowledge question	
Correct answers	92.2%
Incorrect answers	7.8%
COVID-19 need for isolation after positive test basic knowledge question	
Correct answers	93.6%
Incorrect answers	6.4%
COVID-19 form of transmission basic knowledge question	
Correct answers	98.1%
Incorrect answers	1.9%
COVID-19 transmission period basic knowledge question	
Correct answers	83.4%
Incorrect answers	16.6%
Number of correct answers on the COVID-19 basic knowledge questionnaire	
None	0.1%
One	0.3%
Two	4.8%
Three	3.1%
Four	19.6%
Five	72.1%

**Table 5:** Participants’ COVID-19 contact history and symptoms (*n* = 2,903).

Variables	Percentage or median (IQR)
COVID-19 contact history	
Living with a family member that has been infected	
Yes	1.0%
No	6.4%
Haven’t had an infected family member	92.6%
Any co-worker was infected	
Yes	15.2%
No	51.0%
Do not know	33.8%
Close contact with confirmed infected person	
Yes	5.6%
No	46.6%
Do not know	47.8%
Casual contact with confirmed infected person	
Yes	5.6%
No	45.4%
Do not know	49.0%
Any type of contact with people or materials suspected of being infected	
Yes	8.8%
No	33.6%
Do not know	57.6%
Tested for COVID-19	
Yes	4.6%
No	95.4%
COVID-19 symptoms	
Presented at least a symptom in the last 14 days	
Yes	80.6%
No	19.4%
Number of symptoms presented in the last 14 days	
None	19.3%
One	21.2%
Between two and four	47.8%
Between five and seven	10.9%
Between eight and ten	0.8%

**Table 6:** Participants’ COVID-19 risk perception and adhesion to preventive measures (*n* = 2,903).

Variables	Percentage or median (IQR)
COVID-19 risk perception	
General COVID-19 risk perception	10.0 (2.0)
Concern of becoming infected with COVID-19	9.0 (3.0)
Concern about the probability of becoming infected with COVID-19	4.0 (3.0)
Concern about healthcare workers ability to diagnose COVID-19	3.0 (3.0)
Concern about healthcare system ability to diagnose COVID-19	4.0 (3.0)
Concern about the difficulty to treat COVID-19 infection	8.0 (2.0)
Concern about the health consequences of COVID-19 infection	7.0 (4.0)
Concern about the probability of survival if infected with COVID-19	3.0 (3.0)
Concern of transmitting the virus to others	10.0 (0.0)
Risk perception scale total score	60.0 (13.0)
Self-perception of work as a risk for COVID-19 infection	8.0 (7.0)
Belief that had contact with clients/patients that were a risk factor for transmission*	8.0 (7.0)
Acceptance of COVID-19 infection as an occupational hazard	2.0 (6.0)
Belief of avoidance from friends and/or relatives due to working in a high infection risk environment*	
Yes	41.6%
No	40.8%
Do not know	17.6%
Belief that may have contracted COVID-19	
Yes	3.4%
No	40.2%
Do not know	56.4%
Preventive measures	
Using the elbow to cover the mouth while sneezing or coughing	
Never	1.7%
Rarely	2.6%
Sometimes	8.7%
Often	36.3%
Always	50.7%
Avoiding sharing eating utensils during meals	
Never	7.1%
Rarely	4.4%
Sometimes	8.2%
Often	19.2%
Always	61.1%
Washing hands with soap and water	
Never	0.1%
Rarely	0.0%
Sometimes	1.6%
Often	16.8%
Always	81.5%
Washing hands with hydroalcoholic solution	
Never	0.4%
Rarely	2.0%
Sometimes	8.1%
Often	25.5%
Always	64.0%
Washing hands immediately after touching the nose, sneezing or coughing	
Never	2.5%
Rarely	6.2%
Sometimes	19.2%
Often	36.7%
Always	35.4%
Washing hands after touching potentially contaminated objects	
Never	0.2%
Rarely	1.2%
Sometimes	6.9%
Often	26.6%
Always	65.1%
Wearing face mask regardless of symptoms presence	
Never	6.8%
Rarely	4.7%
Sometimes	11.5%
Often	27.1%
Always	49.9%
Keeping a distance of at least a meter and a half from others	
Never	0.7%
Rarely	3.0%
Sometimes	11.5%
Often	27.1%
Always	49.9%
Preventive behaviors scale total score	4.8 (0.6)
Home confinement	
Fully	20.6%
Partially	69.1%
Not confined	10.3%
Social distancing from friends and/or relatives due to perceived higher-risk work environment*	
Yes	84.7%
No	15.3%
Preventive measures effectiveness perception	9.0 (2.0)

*Variables analyzed only within the subset of participants who were not working remotely.

The SOC domains of comprehensibility, manageability and meaningfulness reached a median value of 21.0 (IQR 10.0), 18.00 (IQR 7.0) and 20.0 (IQR 8.0), respectively. And the SOC-13 total scale median score was 58.0 (IQR 21.0) (Table 7).

**Table 7:** SOC-13 individual items and scale scores (*n* = 2,903).

Variables	Median (IQR)
Questions	
Do you have the feeling that you do not really care about what goes on around you?	6.0 (4.0)
Has it happened in the past that you were surprised by the behavior of people whom you thought you knew well?	4.0 (3.0)
Has it happened that people whom you counted on disappointed you?	3.0 (3.0)
Until now your life has had: no clear goals or purpose at all - very clear goals and purpose	6.0 (2.0)
Do you have the feeling that you are being treated unfairly?	5.0 (3.0)
Do you have the feeling that you are in an unfamiliar situation and do not know what to do?	5.0 (3.0)
Doing the things you do every day is: a source of deep pleasure and satisfaction - a source of pain and boredom	5.0 (2.0)
Do you have very mixed-up feelings and ideas?	5.0 (4.0)
Does it happen that you have feelings inside you would rather not feel?	4.0 (4.0)
Many people - even those with strong character - sometimes feel like sad losers in a certain situation. How often have you felt this way in the past?	4.0 (2.0)
When something has happened have you generally found that: you overestimated or underestimated its importance - you saw things in the right proportion	4.0 (3.0)
How often do you have the feeling that there’s little meaning in the things you do in your daily life?	5.0 (3.0)
How often do you have the feeling that you are not sure you can keep under control?	5.0 (3.0)
Scores	
SOC-13 comprehensibility score	21.0 (10.0)
SOC-13 manageability score	18.0 (7.0)
SOC-13 meaningfulness score	20.0 (8.0)
SOC-13 total scale score	58.0 (21.0)

The median UWES-9 total scale score was 3.7 (IQR 2.2), and the domains’ scores varied from 3.0 (IQR 2.3) for vigor to 3.7 (IQR 2.7) and 4.0 (IQR 2.3) for dedication and absorption, respectively (Table 8).

**Table 8:** UWES-9 individual items and scale scores (*n* = 2,903).

Variables	Median (IQR)
Questions	
At my work, I feel bursting with energy	3.0 (2.0)
At my job, I feel strong and vigorous	3.0 (3.0)
I am enthusiastic about my job	3.0 (3.0)
My job inspires me	4.0 (3.0)
When I get up in the morning, I feel like going to work	3.0 (3.0)
I feel happy when I am working intensely	4.0 (3.0)
I am proud on the work that I do	5.0 (3.0)
I am immersed in my work	5.0 (3.0)
I get carried away when I’m working	4.0 (3.0)
Scores	
UWES-9 vigor score	3.0 (2.3)
UWES-9 dedication score	3.7 (2.7)
UWES-9 absorption score	4.0 (2.3)
UWES-9 total scale score	3.7 (2.2)

Finally, 72.6% (95% CI 70.1–74.2%) of the participants presented a GHQ-12 score higher or equal to three and, thus, were classified as being in psychological distress. More than 50% of the participants reported a higher than usual occurrence of: feelings of not being able to overcome difficulties; losing sleep due to worries; feelings of unhappiness and depression; not being able to do enjoy normal day-to-day activities; and feeling constantly under strain (Table 9).

**Table 9:** GHQ-12 individual items and psychological distress prevalence (*n* = 2,903).

Variables	Percentage
Questions	
Have you recently been able to concentrate on whatever you are doing?	
Better than usual	13.8%
Same as usual	43.7%
Less than usual	31.2%
Much less than usual	11.3%
Have you recently lost much sleep over worry?	
Not at all	17.6%
No more than usual	23.5%
Rather more than usual	36.3%
Much more than usual	22.6%
Have you recently felt that you are playing a useful part in things?	
More so than usual	19.7%
Same as usual	45.3%
Less useful than usual	25.2%
Much less useful	9.8%
Have you recently felt capable of making decisions about things?	
More so than usual	9.3%
Same as usual	57.7%
Less so than usual	24.4%
Much less capable	8.6%
Have you recently felt constantly under strain?	
Not at all	8.0%
No more than usual	18.0%
Rather more than usual	41.1%
Much more than usual	32.9%
Have you recently felt you could not overcome your difficulties?	
Not at all	15.0%
No more than usual	32.5%
Rather more than usual	31.9%
Much more than usual	20.6%
Have you recently been able to enjoy your normal day-to-day activities?	
More so than usual	12.3%
Same as usual	26.0%
Less so than usual	38.7%
Much less than usual	23.0%
Have you recently been able to face up to your problems?	
More so than usual	6.9%
Same as usual	51.7%
Less able than usual	31.0%
Much less able	10.4%
Have you recently been feeling unhappy and depressed?	
Not at all	16.1%
No more than usual	22.5%
Rather more than usual	38.1%
Much more than usual	23.3%
Have you recently been losing confidence in yourself?	
Not at all	43.9%
No more than usual	26.7%
Rather more than usual	20.2%
Much more than usual	9.2%
Have you recently been thinking of yourself as a worthless person?	
Not at all	64.1%
No more than usual	16.2%
Rather more than usual	12.5%
Much more than usual	7.2%
Have you recently been feeling reasonably happy, all things considered?	
More so than usual	12.0%
Same as usual	49.8%
Less so than usual	29.1%
Much less than usual	9.1%
Score	
Psychological distress	
Yes (GHQ ≥ 3)	72.6%
NO (GHQ < 3)	27.4%

## 4 Discussion

The present study revealed a high prevalence of psychological distress among Brazilian workers (essential and nonessential) already during the first months of the COVID-19 pandemic. Furthermore, it disclosed that the participants: were mainly working remotely; experienced an increase in workload and in work stress, while being only moderately satisfied with their work; had an adequate COVID-19 basic knowledge; perceived COVID-19 as a serious health problem; and reported being highly adherent to preventive measures.

Regarding the participants’ characteristics, the results show that in general they were young and highly educated white-collar workers, and that almost half of them were civil servants. The majority was married or living with a partner, less than half had children and they predominantly resided in the Southeast region. It should be highlighted that the participants’ sociodemographic and occupational characteristics were quite similar to those of a previous study also conducted among Brazilian workers during the pandemic’s initial phase (57).

The prevalence of psychological distress (GHQ ≥ 3) observed in this study (72.6%) was higher than in other countries where EIQ and the same GHQ-12 cutoff point was used, such as Portugal (57.2%) (58), Peru (59.6%) (59), Argentina (60.9%) (60), Ecuador (62.7%) (47) and Spain (65.1%) (61). Only Chile (78.8%) (62) presented a higher occurrence of psychological distress.

Several factors may explain this prevalence of psychological distress, including the high percentage of remote workers among the participants. Studies performed during the COVID-19 pandemic have already shown that remote work was negatively correlated with psychological distress (34, 61, 63–66). This may have been due to lack of support, isolation, loneliness, low control over long working hours, decreased work productivity and reduced job satisfaction (64, 65, 67). The participants’ gender distribution may also be an explanation, since it has already been shown that women working remotely during the pandemic were more prone to being depressed, anxious, and stressed than men in the same situation (67). Remote work was conceivably over-proportionately burdensome to women, given the unequal distribution of domestic work and family responsibilities dictated by gender roles (63, 64). This issue is particularly pronounced societies that maintain a patriarchal dominance system such as that of Brazil (7, 68, 69), where entrenched gender norms still contribute to the reinforcement of gender roles and inequalities (69, 70). Another work-related factor that may have contributed to the high prevalence of psychological distress observed is the increase in workload reported by the participants, since previous studies have found an association between increased workload and psychological distress (67, 71).

Non-work-related aspects may also be possible explanations for the impressive prevalence of psychological distress found in the study. More than a third (34.1%) of the participants reported having a chronic disease, since it was widely known that many of these conditions presented a greater risk of severe COVID-19 and death in case of infection (72) it is not surprising that a systematic review and meta-analysis showed that chronic diseases patients had the highest prevalence of depression, and high rates of anxiety and distress when compared to the general population, students, healthcare personnel working in clinical departments, workers in non-clinical settings, quarantined individuals and COVID-19 patients (73). Another source of stress for chronic disease patients during the pandemic was the disruption to health services and systems, which caused delay in routine healthcare, treatments interruptions and relationship changes with healthcare workers (74). It is important to note that, numerous studies have highlighted moderate associations between chronic diseases and psychological distress, irrespective of COVID-19. Nevertheless, findings from a twin-paired cross-sectional study indicate that the strength of the association between chronic diseases and psychological distress may be lower than previously presumed (75).

Another interesting result revealed during this study is that the study’s participants presented a median risk perception score of 60 (out of a maximum of 90). On the one hand this is a positive finding, considering that it has already been shown that during epidemic scenarios risk perception is positively associated to preventive measures adherence (76, 77), which was also quite high among the participants. However, on the other hand, higher levels of COVID-19 risk perception were found to be inversely associated with psychological health (78, 79). Thus, it is imperative to set the correct level of risk perception during a pandemic event, in the interest of counterbalancing the adherence to preventive measures and the mitigation of psychological affliction (78, 79).

Improving individuals’ ability to cope effectively with the effects of a pandemic event may be an additional way of dealing not only with the adverse effects of an elevated risk perception but also with psychological distress. According to the salutogenic model, a high level of sense of coherence enables successful coping with regular and acute stressful events (33, 42). This study’s participants presented a lower sense of coherence mean score (*M* = 58.1; SD = 14.7) than those reported by studies (that also made use of the SOC-13) among the adult population of Spain (*M* = 61.6; SD = 12.6) (80) and healthcare workers in Ecuador (*M* = 65.0; SD = 12.7) (38), this might be another mechanism that could contribute to the high prevalence of psychological distress found in this study, since it has already been shown that sense of coherence has a positive strong and significant association with mental health (33).

It is also worthy of note that the workers who took part in this study presented a work engagement mean score (*M* = 3.5; SD = 1.3) similar to that of a study conducted in the United Kingdom (*M* = 3.5; SD = 1.1) (77), but lower than that of an Ecuadorian study (*M* = 4.5; SD = 1.2) (81) and for Spanish healthcare workers (*M* = 4.0; SD = 1.1) (45). Previous studies have shown that psychological distress is inversely associated with work engagement, and that organizations should ensure safe working conditions and promote policies that enable workers to perceive their overall contribution to organization’s goals and foster workers’ development to improve its employees’ work engagement (37). However, even though work engagement has been perceived as a positive worker virtue, it is important to note that more recently it has been shown that over-engagement is associated with burnout (82, 83) and a predictor of exhaustion over time (84) and onset of major depression (85). Thus, it is possible to conclude that work engagement promotion should be done with utmost care, especially during periods of increased psychological distress.

Considering the discussed findings, the evidence indicating that the perception of an adequate governmental response is a determinant of mental health during public health emergencies (29–33), along with the decentralized organizational structure of the Brazilian public health system (which is decentralized and shared by the Ministry of Health and State and Municipal Health Departments), and the political context in Brazil during the studied period, it is reasonable to presume that a coordinated, evidence-based pandemic response led by the Ministry of Health would likely reduce the prevalence of psychological distress among Brazilian workers. It is crucial to bear in mind that, between April and May 2020 Brazil witnessed three changes in health ministers, epidemiological data from the Ministry of Health was unreliable and lacked transparency, while federal government coordination of the pandemic response was nearly non-existent (15–20). Additionally, there was a consistent downplaying of the COVID-19 risk by high-ranking federal government members, who not only opposed state-mandated social distancing measures but also criticized state governors’ decisions to implement restrictions. They actively promoted drugs like hydroxychloroquine and ivermectin, known to be ineffective against COVID-19, while discouraging the use of face masks (15–20).

It is important to interpret this study’s results while considering its limitations. Despite GHQ-12’s widespread use in cross-cultural comparisons, evidence of measurement equivalence across its different language versions are still lacking. Therefore, from a stringent psychometric perspective, caution is advised in interpreting mean differences between countries as indicative of distinct levels of psychological distress (86). This is due to the inability to ascertain whether such differences genuinely reflect variations in psychological distress or are instead attributable to inherent measurement issues (86).

Regardless of its potential for biased estimates, snowball sampling was employed in this study. This sampling strategy may have limited participant representativeness, as indicated by the high percentage of female participants and of those with at least bachelor’s degrees. Another potential bias related to the snowball sampling method involves the referral of individuals which tend to have similar beliefs, values, and attitudes, possibly introducing high uniformity. This could result in an unknown and immeasurable, although identifiable, selection bias in the data. Additionally, it is known that individuals with existing or severe mental illness are less likely to participate in online research than those without such conditions (87). Therefore, even though our results findings might be valuable, it is possible that they still underestimate the actual extent of psychological distress among Brazilian workers. It is worth mentioning that most of the published studies that assessed psychological distress during the COVID-19 pandemic made use of snowball sampling. It is also noteworthy that, to reduce selection bias from snowball sampling, the researchers purposefully selected from their professional and social network well-connected individuals with diverse education, socioeconomic and working backgrounds as initial seeds. It was also asked from this first wave of recruits to contact only three to five new recruits in the subsequent wave and so forth, to prevent those with larger social networks from dominating the sample. However, it was not possible to track social ties and collect information on participants’ network sizes.

Finally, like other numerous studies conducted during the COVID-19 pandemic, data collection was online, and thus limited to individuals with internet access. Even though 79.5% of Brazilian households had internet access in 2019 (88), those of the lowest income and educational groups, which were likely to differ in many ways from the study’s participants, may have been excluded. It should be highlighted that, there are evidences indicating few differences between research data collected online and those obtained through traditional self-report methods, as well as those participants recruited online may be demographically diverse and equally motivated to provide reliable data (49, 89). It should be clear that none of these dismiss the disadvantages imposed by snowball sampling discussed above. Nevertheless, it is important to note that both limitations, snowball sampling and online data collection, were imposed by legal (and ethical) issues associated with the need for containing COVID-19 transmission.

Despite the above limitations, some of the major strengths of the present study were the large and geographically distributed sample obtained, the use of internationally validated instruments, and the fact that the same research questionnaire was used in several countries making it possible to compare with caution our findings with those obtained in other nations.

In conclusion, a total of 2,903 Brazilian workers from diverse work sectors participated in the study. The study’s participants presented a lower sense of coherence and work engagement than those observed in previous studies. Regarding the main outcome, almost three quarters of respondents were classified as presenting psychological distress. Therefore, the provision of remotely delivered mental health interventions for workers during the early stages of public health events that necessitate prolonged social distancing measures may be helpful to maintain mental health. Many of such interventions have been developed and implemented over the COVID-19 pandemic, now is the time to evaluate their feasibility and effectiveness in different settings, in such a way that all countries should be able to prepare emergency plans that include tools to better cope with mental health problems during future pandemics, minimizing economic, social and health consequences.

Although the present study provides valuable information that may aid in laying the groundwork for targeted interventions and policy recommendations throughout the early stages of a future pandemic, there remains a need for research that assesses the factors associated with psychological distress, the long-term effects of the COVID-19 pandemic on the mental health of essential and non-essential workers, and the effectiveness and safety of interventions aimed at preserving mental health, strengthening the sense of coherence, and promoting work engagement among working populations during a pandemic event.

## Data availability statement

The original contributions presented in the study are included in the article/supplementary material, further inquiries can be directed to the corresponding author.

## Ethics statement

The study was authorized by the Brazilian National Research Ethics Committee (CAAE 30437120.4.0000.5411, 04/23/2020). It was conducted in accordance with the local legislation and institutional requirements. The participants provided their written informed consent to participate in this study.

## Author contributions

MA: Conceptualization, Data curation, Formal analysis, Investigation, Methodology, Project administration, Resources, Software, Supervision, Validation, Visualization, Writing – original draft, Writing – review & editing. ML: Conceptualization, Data curation, Formal analysis, Investigation, Methodology, Resources, Software, Supervision, Validation, Visualization, Writing – original draft, Writing – review & editing. AD: Conceptualization, Data curation, Formal analysis, Investigation, Methodology, Resources, Software, Supervision, Validation, Visualization, Writing – original draft, Writing – review & editing. JC-V: Conceptualization, Data curation, Formal analysis, Investigation, Methodology, Resources, Software, Supervision, Validation, Visualization, Writing – original draft, Writing – review & editing. JG-I: Conceptualization, Data curation, Formal analysis, Investigation, Methodology, Resources, Software, Supervision, Validation, Visualization, Writing – original draft, Writing – review & editing. CR-F: Conceptualization, Data curation, Formal analysis, Investigation, Methodology, Resources, Software, Supervision, Validation, Visualization, Writing – original draft, Writing – review & editing. JB: Conceptualization, Data curation, Formal analysis, Investigation, Methodology, Resources, Software, Supervision, Validation, Visualization, Writing – original draft, Writing – review & editing. JG-S: Conceptualization, Data curation, Formal analysis, Investigation, Methodology, Resources, Software, Supervision, Validation, Visualization, Writing – original draft, Writing – review & editing.
